# Effects of applied potential and the initial gap between electrodes on localized electrochemical deposition of micrometer copper columns

**DOI:** 10.1038/srep26270

**Published:** 2016-05-17

**Authors:** Fuliang Wang, Hongbin Xiao, Hu He

**Affiliations:** 1 State Key Laboratory of High Performance Complex Manufacturing, Changsha 410083, China; 2 School of Mechanical and Electrical Engineering, Central South University, Changsha 410083, China

## Abstract

Micrometer copper columns were fabricated via a technology named localized electrochemical deposition (LECD). This paper studies the effects of applied potential and the initial gap between electrodes on the LECD process. The surface and cross sectional morphologies, as well as the average deposition rate were investigated to evaluate the quality of the deposited copper columns. Results demonstrated that the copper columns tended to be cylinder-shape with few voids inside at lower potential (<2.4 V). Whereas,the copper columns tended to be dendriform-shape with lots of voids inside at larger potential (>2.8 V). The average deposition rate increased with the raise of potential. In addition, the copper columns tended to be cylinder-shape with the initial gap between electrodes to be 10 *μm* or below. However, the copper columns tended to be cone-shape when the initial gap between electrodes became larger (35 *μm* or above). The number of voids inside the copper column and the average deposition rate both decreased with the increase of the initial gap. Moreover, the process of LECD under varied electric field has also been simulated using COMSOL software, and the formation of cylindrical and conical copper columns was further explained based on the electric field distribution at the cathode.

Since the method of localized electrochemical deposition (LECD) was proposed by Hunter *et al*. in 1995, it has rapidly been a rising technology for fabricating three-dimensional microstructures after etching and micromachining^1^,^2^,^3^. The method of LECD can fabricate intensive structures with high aspect ratio and complicated geometry. Given that this method employs micro-anode guided electroplating (MAGE)^4^ in the open air, high-end equipment and absolutely clean room are not required^5^,^6^, which makes this method simpler, cheaper and cleaner than conventional electroplating approaches. Moreover, it has demonstrated that various materials can be deposited by LECD, including metals, metal alloys, conducting polymers and even some semiconductor materials^7^,^8^,^9^,^10^.

In order to investigate the mechanism of localized electrochemical deposition, Seol *et al*.^11^,^12^ developed a real-time technique of micro-radiology by means of coherent X-rays to monitor the LECD process *in situ*. They proposed a qualitative interpretation for the interplay between metal-ion diffusion and migration in the deposition process. El-Giar *et al*.^13^ found out that the electrical potential, the concentration of the copper sulfate solution and the presence of organic additives all can affect the microstructure of the deposits as well as the current efficiency of the electroplating process. They also proposed the optimum range of the applied voltage and the concentration of the copper sulfate solution as well as the organic additives to form micro-copper columns with fine grain, smooth surface and compact structures. Lin *et al*.^14^ pointed out that LECD process under pulse current (PC) mode was better than that under direct current (DC) mode. Lee *et al*.^15^ employed an anode with higher strength to withstand the cavitation effect caused by the generated gas bubbles at the end tip and prevent the direction of deposition changing from vertical to horizontal. As a result, the densification of the deposited copper columns improved significantly. In addition, other methods have also been proposed to change the condition of electroplating. Yeo *et al*.^16^ reported that ultrasonic vibrations can increase the deposition rate and improve the concentricity of the fabricated micro-columns in the LECD process, while increase the porosity of the deposited micro-columns. Their later work^17^ pointed out that deposition with electrode rotation produced columns with annulus cross sections, which indicated the existence of a uniform hollow core inside the columns. Surprisingly, very few works have been done to elaborately explore the effect of the experimental parameters in the LECD process on the fabrication of micrometer copper columns. Additionally, few works were devoted to the effect of the electric field distribution on the surface of cathode to the LECD process so far. However, we believe that well understanding the effect of the critical experimental parameters, such as the applied potential, the initial gap and electric field distribution, to the LECD process, would be very significant to widely apply LECD process for fine and complex micro-structure fabrication.

In this paper, the potential and the initial gap between electrodes were adjusted during the LECD process to investigate the structural changes of the micrometer copper columns. In addition, the process of LECD has also been simulated using COMSOL Multiphysics (COMSOL for short) software and the electric field distribution was analyzed to explain the mechanism towards the different surface morphologies of micrometer copper columns.

## Results

### Effects of the applied potential on the surface morphologies,cross sectional morphologies and average deposition rate of the copper columns

**Figure 1:**
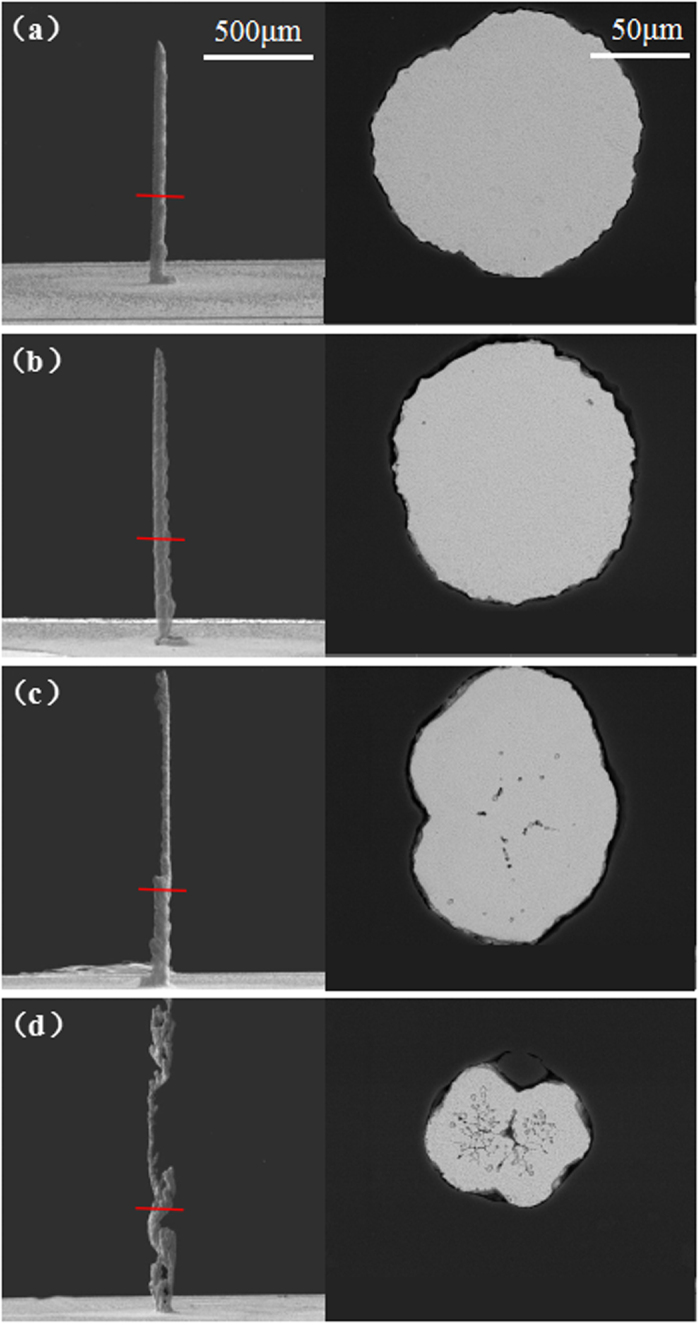
SEM morphologies for the micrometer copper columns deposited at different potentials and their corresponding cross sections. The copper columns were deposited at (**a**) 2.4 V, (**b**) 2.6 V, (**c**) 2.8 V and (**d**) 3.0 V with the initial gap between electrodes of 5 *μm*.

Figure 1 displays the SEM images of the copper columns’ surface morphologies and their corresponding cross sections at the position that marked with a line across the copper columns deposited at the potentials of 2.4 V, 2.6 V, 2.8 V and 3.0 V, respectively. It can be seen that both the surface and the cross sectional morphologies varied significantly at different potentials. When potential is 2.4 V, the copper column tends to be cylinder-shape with uniform diameter and the shape of the cross section is approximate to be a circle with few voids inside. As the potential increases to 2.6 V, tumors begin to appear on the copper column. The bifurcate phenomenon of the deposited copper column appears at the potential of 2.8 V and the shape of cross section becomes irregular with some voids inside. Additionally, once the potential reaches 3.0 V, the copper column is formed in dendriform-shape and the cross section is full of voids and cracks.

**Figure 2:**
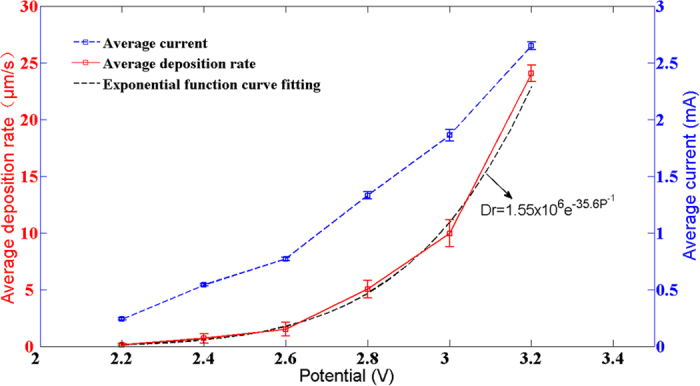
A plot of the average current and average growth rate for the columns against the applied potential employed in the LECD.

Average deposition rate is calculated by dividing the total height of copper column by the deposition time. Figure 2 depicts a plot of the average current and average growth rate for the columns against the applied potential employed in the LECD, which indicates the average deposition rate increases with the potential. When the potential is 2.2 V, the average deposition rate is close to zero. However, as the potential rises to 3.2 V, the average deposition rate increases to 23 *μm/s*. An exponential function that fits the relationship between potential and average deposition rate is expressed as


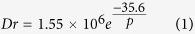


where *Dr* represents the average deposition rate, *p* represents the applied potential. Thus, the average deposition rate increases exponentially when potential is in the range of 2.2 V to 3.2 V. This fitted model could be employed to predict the average deposition rate under other applied potential. In addition, as shown in Fig. 2, the average current is linear proportional to the applied potential, which indicates the relationship between the observed average current and the deposition rate is similar to that between the applied potential and the deposition rate.

### Effects of the initial gap between electrodes on the surface morphologies,cross sectional morphologies and average deposition rate

**Figure 3:**
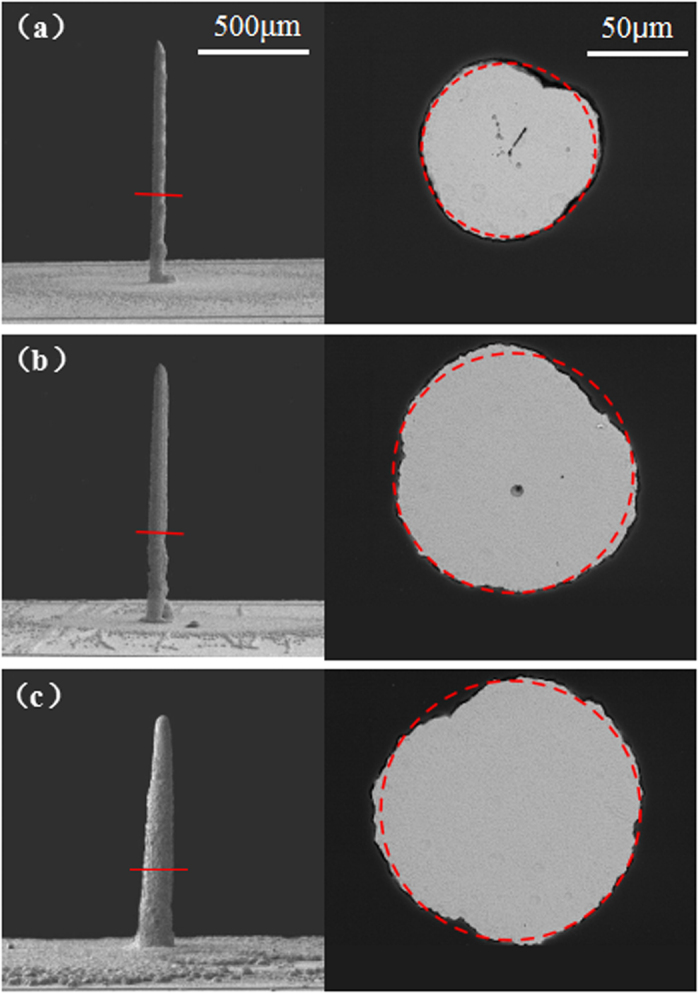
SEM morphologies for the micrometer copper columns deposited at different initial gaps between electrodes and their corresponding cross sections. The copper columns were deposited at the initial gap of (**a**) 5 *μm*, (**b**) 15 *μm* and (**c**) 35 *μm* with the potential of 2.4 V.

The SEM images of the surface and the corresponding cross sectional (the position of a copper column marked by a line) morphologies of the copper column deposited with the initial gaps as 5 *μm*, 15 *μm* and 35 *μm*, respectively, are shown in Fig. 3. It can be seen that when the initial gap between electrodes is 5 *μm*, the copper column tends to be cylinder-shape and there are some voids inside the cross section. When the initial gap between electrodes increases to 15 *μm*, the copper column is still cylinder-shape, but the number of voids inside the cross section decreases apparently. When the initial gap is set as 35 *μm*, the copper column tends to be cone-shape with diameter decreasing from the bottom to the top and there are nearly no voids inside the cross section.

**Figure 4:**
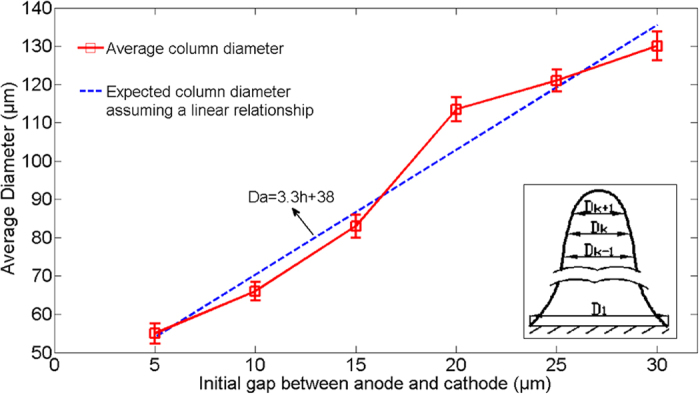
A plot of average diameter of the copper column against the initial gap between electrodes. The inset illustrates the way to compute the average diameter for single deposited micro-column.

Considering that the deposited micro-column is not perfect cylinder shape. Thus, we computed the average diameter for each column firstly as shown in the inset of Fig. 4. If we define the diameter of arbitrary cross section of the copper column as *D*_*k*_, the average column diameter (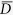) for each deposited column can be then expressed as:


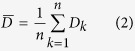


where *n* is set to be 20 in our experiment. Then, the final average diameter was obtained using four columns under the same initial gap condition. Figure 4 depicts a plot of average diameter of the copper columns against the initial gap between electrodes, which indicates the average diameter increases linearly with the initial gap between electrodes. When the initial gap increases from 5 *μm* to 30 *μm*, the average diameter of the column changes from 56 *μm* to 130 *μm*. A linear function is used to fit the relationship between the initial gap and the average diameter of the columns as follows:


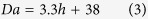


where *Da* represents the average diameter of the columns and *h* represents the initial gap between electrodes.

**Figure 5:**
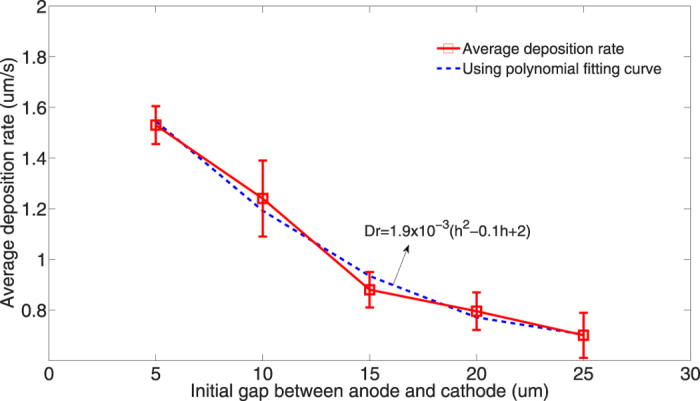
A plot of average deposition rate against the initial gap between electrodes.

Average deposition rate of LECD is calculated in the same way as mentioned before, i.e., dividing the total height of copper column by the deposition time. Figure 5 depicts the relationship of the average deposition rate against the initial gap between electrodes. It can be seen that the average deposition rate decreases as the initial gap between electrodes increases. When the initial gap increases from 5 *μm* to 25 *μm*, the average deposition rate decreases from 1.53 *μm/s* to 0.7 *μm/s*. A quadratic function is used to fit the relationship between the initial gap and average deposition rate:


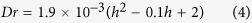


where *Dr* represents the average deposition rate and *h* represents the initial gap between electrodes. This fitted model could be employed to predict the average deposition rate at specific initial gap.

## Discussion

### The influencing mechanism of potential in the process of LECD

As shown in Figs 1 and 2, potential has a great effect on the surface morphology and average deposition rate. The lower potential would suppress the deposition rate, which means copper ions can be diffused to the LECD region and supply the consumed copper ions timely. Therefore, when the deposition occurs at the potential under the critical value (~2.6 V in our experiment), the deposited copper columns tend to be compact structure, smooth surface and regular shape. Nevertheless, when the potential is higher than 2.6 V, the deposition rate is much faster, which makes copper ions difficult to be diffused timely to supply the consumed copper ions in the LECD region. Thus, the Cu columns tend to be tumourful, bifurcate and dendriform shape^13^,^18^.

**Figure 6:**
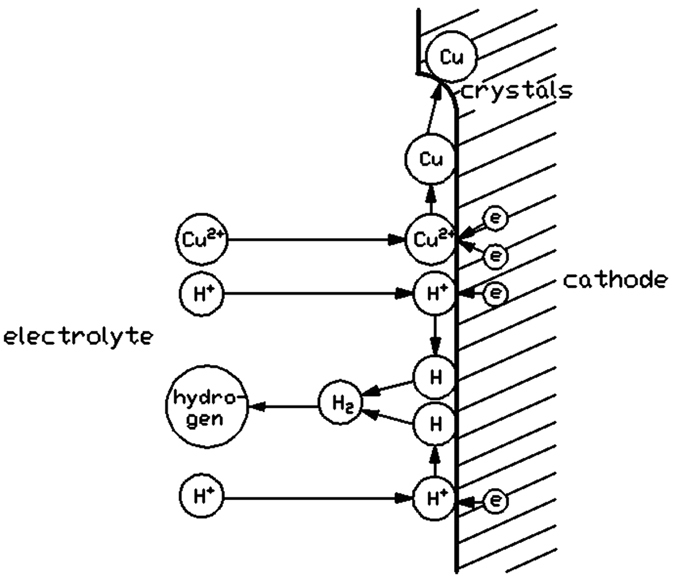
The reduction process of copper ions and hydrogen ions on the surface of cathode during the LECD process.

As shown in Fig. 1, potential can also affect the formation of voids inside the copper columns. During the LECD process, there are two major reactions on the surface of cathode. One of the reactions is that copper ions transform into copper atoms by absorbing electrons. The other is the hydrogen evolution reaction, that is, hydrogen ions transform into hydrogen atoms by absorbing electrons. These hydrogen atoms will then combine to become hydrogen. The reduction process of the two kinds of ions on the surface of cathode is shown in Fig. 6. Hydrogen forms some bubbles inside the fluid after separating out from the cathode and they always attach on the surface of cathode. These bubbles will then hinder the deposition of copper ions at the place. If the hydrogen bubbles stay at the surface of cathode all the time in the process of electroplating, it will result in voids or penetrating crack inside the columns. The solution in the vicinity of the substrate (beneath the micro-anode) becomes depleted in the ions required for discharge at higher current, and if the current exceeds the limiting value for a given electrolyte, large numbers of hydrogens will be evolved at the same time as the copper is deposited, which introduces more voids in the deposited copper column^13^. As the applied potential is proportional to the current, more voids will then form inside the deposited copper column at larger potential.

### The effect of electric field distribution on the surface of cathode in the process of LECD

**Figure 7:**
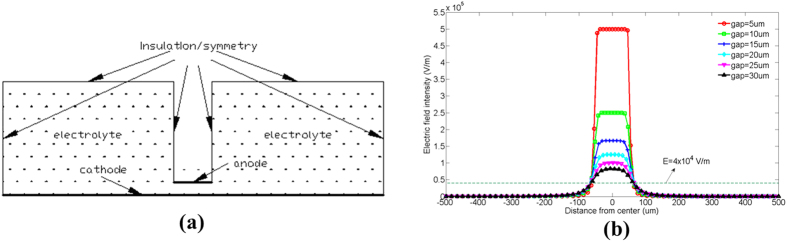
(**a**) The planar model built in the simulation with COMSOL. (**b**) The electric field distribution on the surface of cathode at the initial time (0 s) in the first stage of electroplating under different initial gaps with the potential as 2.4 V in the simulation.

To understand the electric field distribution on the surface of cathode, the process of LECD is simulated using COMSOL. The planar model is established as shown in Fig. 7(a). In the model, the width of anode, the width of cathode and the height of the electrolyte are 100 *μm*, 1000 *μm* and 300 *μm*, respectively.

Since the micro-anode moves upward intermittently in the experiment, the process of electroplating is corresponding intermittent. The process of deposits growing from the Ag substrate to the height of *h* is defined as the first stage of electroplating. Likewise, the process of deposits growing from the height of (n−1)*h* to the height of *nh* is defined as the n^th^ stage of electroplating. The electric field distribution around the surface of cathode at the initial time (0 s) in the first stage of electroplating under different initial gaps is simulated as shown in Fig. 7(b). It can be seen that the electric field intensity reaches to the maximum in the area near to the center of cathode and decreases rapidly at a distance away from the center. Moreover, all these curves in the figure nearly intersect at two symmetrical points. The electric field intensity for the two points is about 4 × 10^4^ V/m, and the corresponding width at the cathode is about 150 *μm* which is close to the bottom diameter of the copper column deposited at the potential of 2.4 V in the experiment. Therefore, there could be a critical electric field intensity^19^ with the value of about 4 × 10^4^ V/m. When the electric field intensity is lower than this threshold, the deposition will not happen.

**Figure 8:**
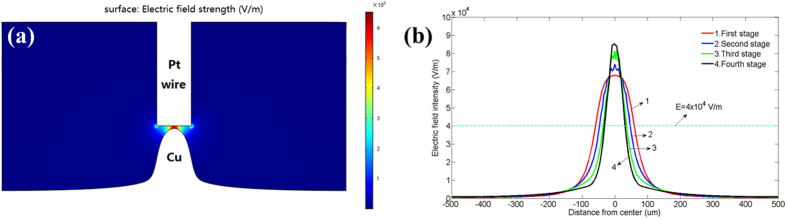
(**a**) Distribution map of the electric field strength of the whole electroplating domain simulated with the software COMSOL. (**b**) The electric field distribution on the surface of cathode at the initial time (0 s) from the first to the fourth stage of electroplating. The potential is 2.4 V and the initial gap between electrodes is 35 *μm*.

**Figure 9:**
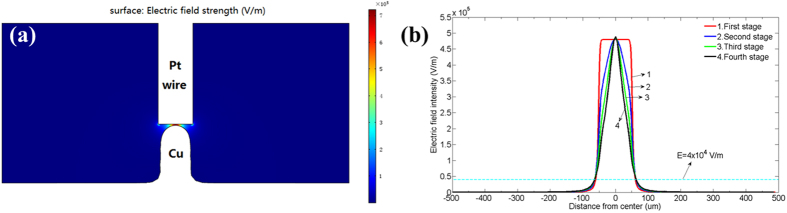
(**a**) Distribution map of the electric field strength of the whole electroplating domain simulated with the software COMSOL. (**b**) The electric field distribution on the surface of cathode at the initial time (0 s) from the first to the fourth stage of electroplating. The potential is 2.4 V and the initial gap between electrodes is 5 *μm*.

As shown in Fig. 3, when the initial gap exceeds 15 *μm*, the copper column tends to be a cone. While the initial gap is less than 15 *μm*, the copper column tends to be a cylinder. To understand the relationship between electric field and the shape of copper column, the variation of electric field distribution on the surface of cathode during the process of LECD is obtained through the simulation. When the initial gap between electrodes is 35 *μm*, the distribution map of the electric field strength of the whole electroplating domain and the electric field distribution on the surface of cathode at the initial time (0 s) from the first to the fourth stage of electroplating in the simulation is shown in Fig. 8. It can be seen that the electric field intensity reaches to the maximum in the area near to the center of cathode and decreases rapidly at a distance away from the center. Moreover, the electric field intensity of the earlier stage is smaller than that of the latter stage and the width with electric field intensity over the threshold (4 × 10^4^ V/m) continuously narrows down, which indicates that the diameter of copper column deposited at the cathode will become smaller and smaller during the process of electroplating. Hence, this may explain the formation of conical copper columns in the experiment. When the initial gap between electrodes decreases to 5 *μm*, the distribution map of the electric field strength of the whole electroplating domain and the electric field distribution on the surface of cathode at the initial time (0 s) from the first to the fourth stage of electroplating in the simulation are shown in Fig. 9. It can be seen that the width of electric field intensity exceeding the threshold (4 × 10^4^ V/m) nearly keeps the same, which means that the diameter of the copper column deposited at the cathode will not change during the process of electroplating. Hence, this may explain the formation of cylindrical copper columns in the experiment.

## Conclusion

The effects of the applied potential and the initial gap between electrodes on the surface morphology and deposition rate for the deposited copper column are investigated in this paper. It demonstrates that copper columns with regular shape and compact structures are fabricated at low potential. When potential becomes higher, the copper column tends to be dendriform-shape. The average deposition rate increases with the raise of potential. The copper columns tend to be cylinders when the initial gap between electrodes is small, whereas the copper columns tend to be cone-shape when the initial gap becomes larger. The average deposition rate decreases with the raise of the initial gap between electrodes.

According to the simulation by COMSOL, we found that the diameter of copper column is affected by electric field distribution at the cathode. When the initial gap between electrodes is quite large, the width with electric field intensity over the threshold (4 × 10^4^ V/m) narrows down continuously. When the initial gap between electrodes is small, the width with electric field intensity over the threshold (4 × 10^4^ V/m) nearly keeps the same. Based on the difference of electric field distributions at the cathode, the formation of cylindrical and conical copper columns in the experiment could be explained.

## Methods

A platinum wire (100 *μm* in diameter) insulated in a capillary glass tube filled with epoxy resin was used as the anode. The cathode was made of an Ag-coated Cu substrate (6 mm × 4.5 mm × 0.1 mm) mounted with epoxy resin. The front surface of the cathode plate was ground by a sequence of emery papers and wetly polished to reveal a mirror surface^20^. The electrolyte used in this study was composed of 0.8 M copper sulfate (CuSO_4_·5H_2_O) at room temperature.

**Figure 10:**
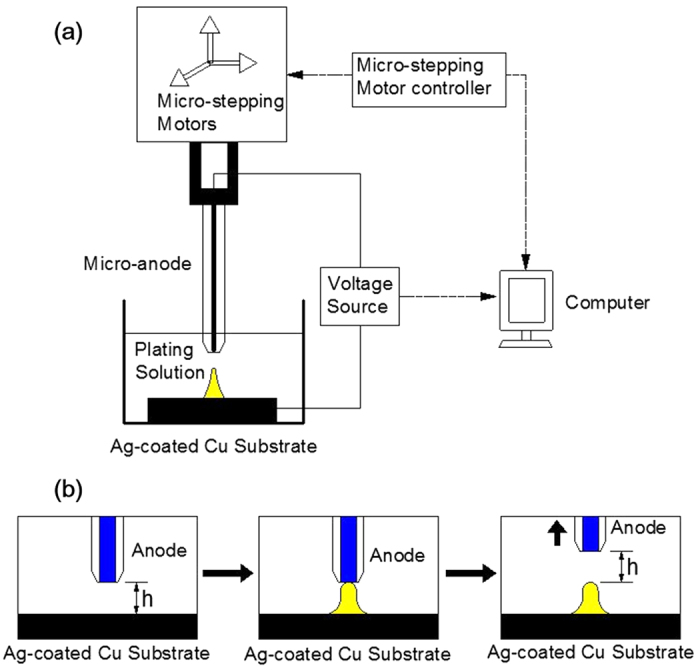
(**a**) Schematic of the experimental setup for localized electrochemical deposition. (**b**) The sequential diagrams to show the growth of a micrometer copper column in the LECD process.

Figure 10 depicts the schematic of the experimental setup for localized electrochemical deposition and sequential diagrams to show the growth of a micrometer copper column in the LECD process. The micro-anode was driven by a micro-stepping motor to move upward intermittently and controlled by a computer interface. Since the micro-anode moves upward intermittently with the same height each time, the electroplating process is also repeated intermittently. In the beginning of each cycle, the micro-anode was in contact with the cathode. Before the electroplating started, the micro-anode moved upward under the control of micro-stepping motors to form an initial gap as *h* between the tip of micro-anode and the top surface of the cathode. When the process of electroplating began, the copper column continued growing from the Ag substrate. Once the top of the growing copper column touched the tip of the micro-anode, the micro-anode moved upward to obtain another gap distance of *h*.

A scanning electron microscope (SEM) (TESCAN MIRA3 LMU) was used to examine the surface morphology and microstructure of the micrometer copper columns. To observe the cross section of the copper column, the column was embedded into an epoxy resin and then polished to a mirror-like surface using a series of emery papers and the slurries of fine alumina.

## Additional Information

**How to cite this article**: Wang, F. *et al*. Effects of applied potential and the initial gap between electrodes on localized electrochemical deposition of micrometer copper columns. *Sci. Rep.* **6**, 26270; doi: 10.1038/srep26270 (2016).
